# Electrohydraulic lithotripsy through endoscopic retrograde cholangiopancreatography combined with SpyGlass in the treatment of complex pancreatic duct stones: A case report and literature review

**DOI:** 10.3389/fsurg.2023.1059595

**Published:** 2023-01-18

**Authors:** Weigao Pu, Chenhui Ma, Bofang Wang, Yunpeng Wang, Haiyun Wang, Bo Xu, Puyi He, Hongbin Cui, Hao Chen

**Affiliations:** ^1^ The Second Clinical Medical College, Lanzhou University, Lanzhou, China; ^2^ Department of Tumor Surgery, Lanzhou University Second Hospital, Lanzhou, China; ^3^ Department of Science and Technology, Key Laboratory of Digestive System Tumors of Gansu Province, Lanzhou, China

**Keywords:** pancreatic duct stones, SpyGlass, endoscopic retrograde cholangiopancreatography, electrohydraulic lithotripsy, pancreatitis

## Abstract

The incidence of pancreatic duct stones (PDS) is less than 1%. After the formation of stones, the lumen of the pancreatic duct is blocked, and the pancreatic juice cannot be discharged smoothly, resulting in the impairment of the internal and external secretions of the pancreas. Several national guidelines now recommend endoscopic retrograde cholangiopancreatography (ERCP) as the treatment for PDS. The emergence of SpyGlass makes it possible to visualize the ERCP blind area of the pancreatic system directly. Electrohydraulic lithotripsy (EHL) under SpyGlass can crush large and pressure-resistant stones into smaller fragments, significantly improving the success of the endoscopic treatment of large stones. Here, we report a patient presented with acute alcohol-associated pancreatitis, found to have PDS on imaging, who underwent ERCP combined with SpyGlass (EHL), avoiding surgery, reducing trauma, and being discharged from the hospital with a rapid recovery. Therefore, endoscopic therapy is effective and safe for PDS patients. The combination therapy of this patient is the first use of SpyGlass for PDS in our centre, which marks a new stage in the application of endoscopic therapy for pancreatic diseases.

## Introduction

The incidence of PDS does not exceed 1% in the whole global population (1). PDS are often secondary to chronic pancreatitis (CP). Over time, their incidence reaches 50% to 100% in the fifth and fourth years of CP (2). Surgical treatment of PDS has significant trauma, many complications, and a long recovery time, and endoscopic treatment reduces unnecessary surgical operations (3). However, treating PDS with ERCP alone has always been a great challenge. The application of SpyGlass makes ERCP move towards direct visualization, which can reduce the frequency of angiography (4). Electrohydraulic lithotripsy (EHL) or laser lithotripsy (LL) under SpyGlass can crush large and pressure-resistant stones into smaller fragments, significantly improving the endoscopic treatment of large stones, and appears to be a good alternative and a very successful therapeutic approach. These combinatorial treatment strategies yield great success rates and guarantee safety (5).

Here, we provide a case report of a patient who suffered from nausea, vomiting and severe abdominal pain after drinking alcohol. The patient underwent a CT scan showing a large calculus (1.5 cm*1.2 cm) in the pancreatic duct and head. After the multidisciplinary team (MDT) discussion, it was decided to use ERCP combined with SpyGlass minimally invasive treatment. Using EHL, the stone was successfully crushed, no postoperative complications occurred, and there was no recurrence of PDS during the 1-year follow-up. Therefore, we demonstrate that ERCP combined with SpyGlass can crush and remove stones under direct vision. The procedure improved the patient's quality of life, confirming that this minimally invasive treatment of PDS is effective and safe.

## Case presentation

**Figure 1:**
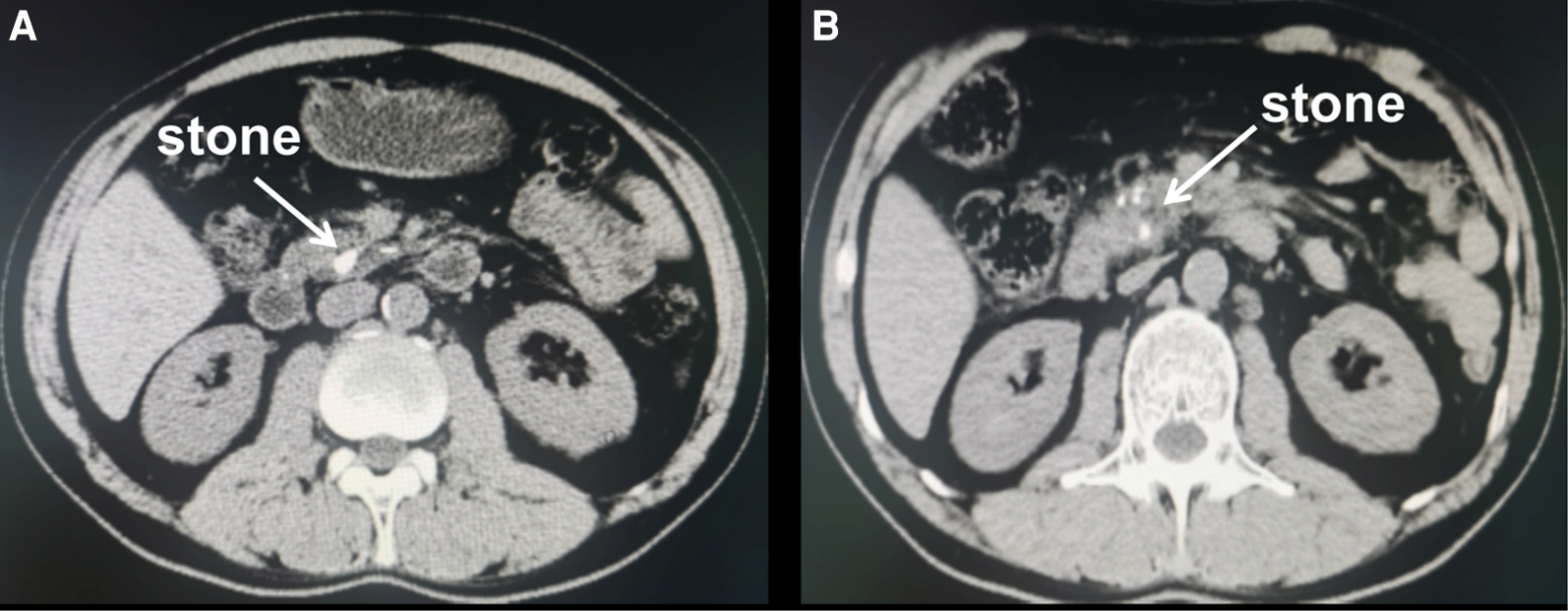
Computed tomography scan of the patient's abdomen. (**A,B**) CT showing acute pancreatitis and large calculi in the pancreatic duct (white arrow).

A 54-year-old male with severe abdominal pain after alcohol consumption for more than 20 days, accompanied by epigastric pain, nausea and vomiting with a history of alcohol abuse, was enrolled in the case study. The patient was admitted to the Second Hospital of Lanzhou University and was diagnosed with acute pancreatitis after abdominal computed tomography (CT) scan examinations showed that CT imaging was suggestive of large stones in the head of the pancreatic duct and dilation of the entire main and branch pancreatic duct. Furthermore, multiple calcifications in the pancreas were also detected (Figures 1A,B). In addition, routine blood tests showed white blood cells 15.18 × 10^9^/L↑, blood amylase (U/L) 650↑, serum lipase (U/L) 881↑, IL-6 (pg/ml) 89.11↑, and procalcitonin (ng/ml) 0.637↑. ERCP combined with SpyGlass (model: iMES-I-D; Electrode: 0.9 mm in diameter, 3.75 m in length. Boston Scientific, Marlborough, Massachusetts, USA) was used to extract and remove the large stone. Intravenous general anaesthesia was applied, and the patient took a left lateral position. The endoscopic procedure went on smoothly, inserted *via* the oesophagus and the gastric cavity, which went through and passed the duodenal bulb without any hindrances.

**Figure 2:**
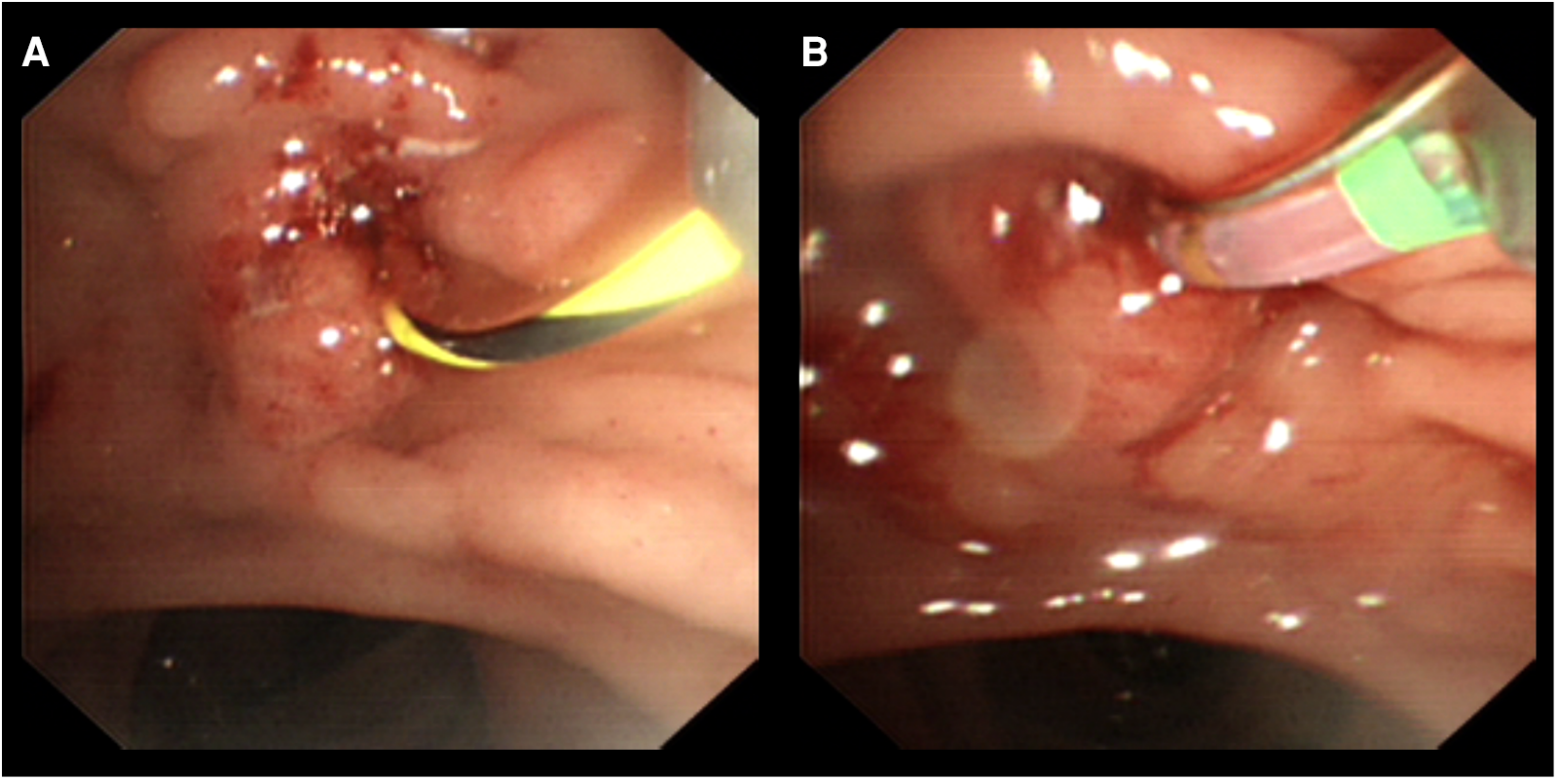
ERCP progress. (**A,B**) Cannulation through the duodenal bulb to the main pancreatic duct under the guidance of the guidewire.

**Figure 3:**
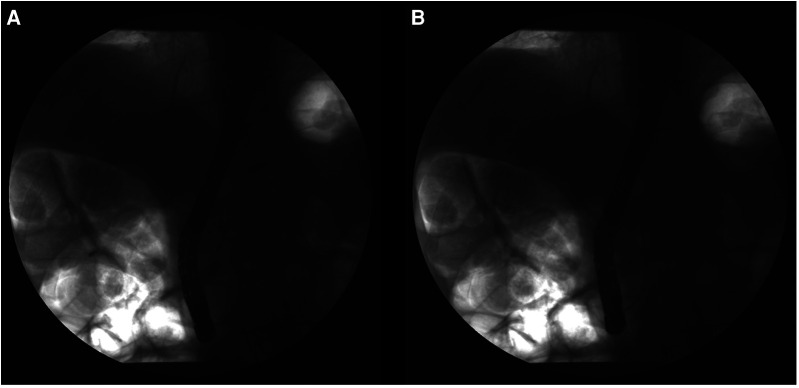
Guide wire is inserted into the pancreatic duct, and a contrast agent is injected. (**A**) The guide wire was introduced to the pancreatic duct, multiple irregular filling defects in the pancreatic duct can be seen, and the pancreatic duct cavity appears narrow; (**B**) Cylindrical balloon dilatation for approximately 30 s.

**Figure 4:**
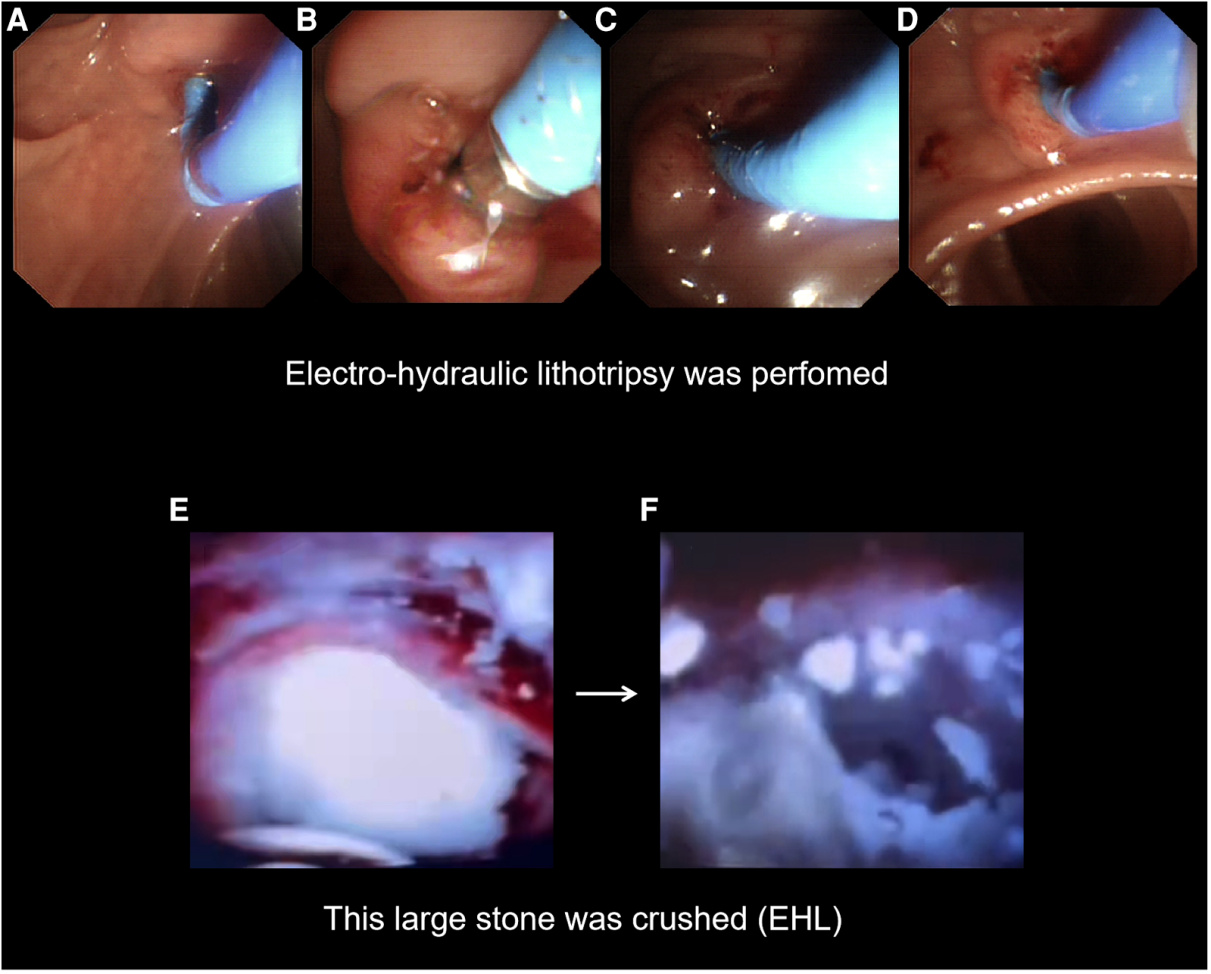
Insert the SpyGlass into the pancreatic duct and operate. (**A–D**) Insertion of the SpyGlass into the pancreatic duct represents a view through the SpyGlass direct visualization system, which shows a large white stone in the main pancreatic duct. (**E,F**) Insertion of the U100 Plus EHL lithotripsy fibre through the working pipe to the surface of the stone for electrohydraulic lithotripsy was carried out.

**Figure 5:**
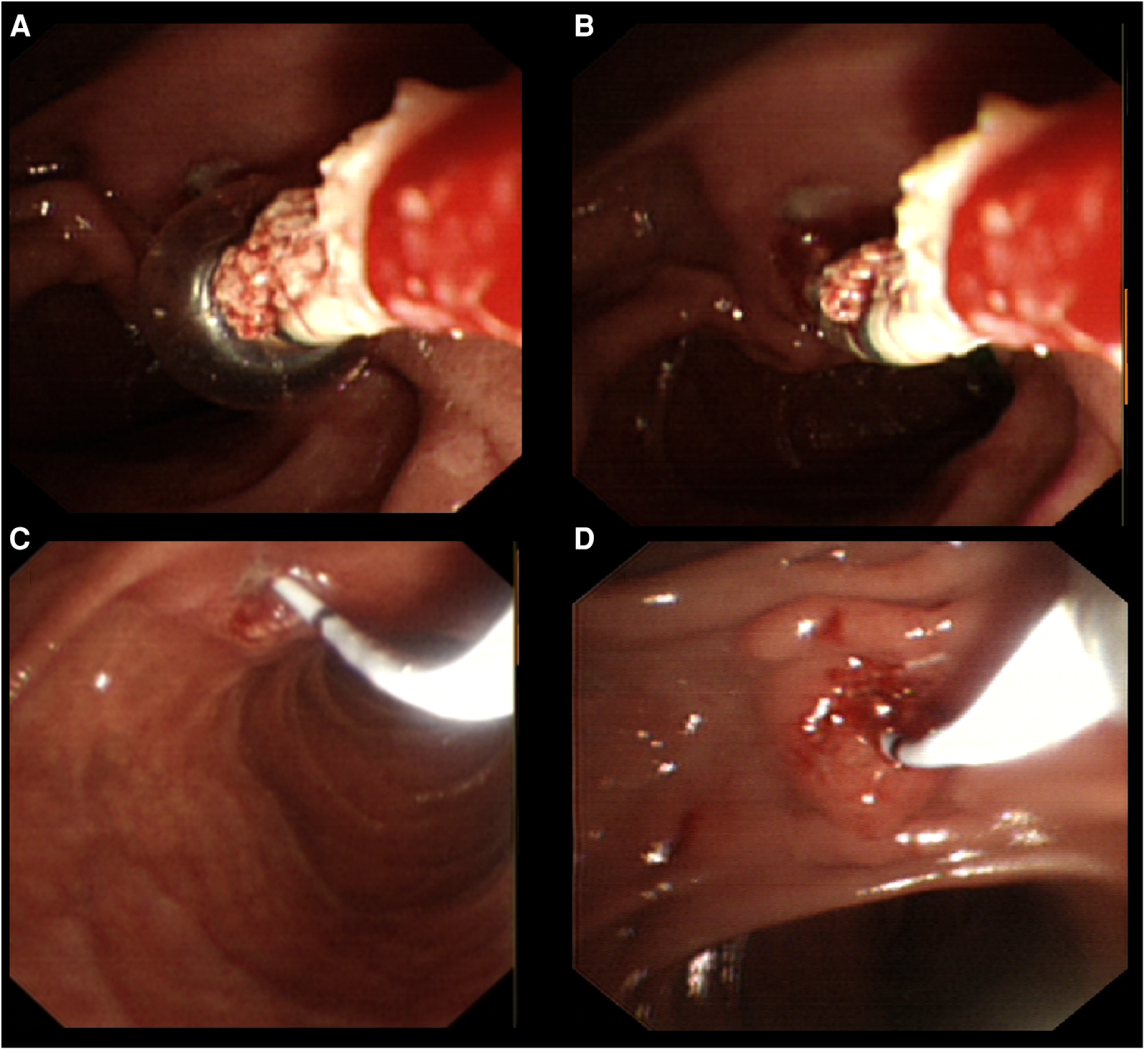
Stone removal process. (**A,B**) Removal of the stone of the pancreatic duct by a balloon. (**C,D**) Placement of a minimally invasive pancreatic duct pigtail stent.

The observation took the distance from the duodenum to the major papilla of the duodenum, which is a hemispherical bulge, and then to the accessory papilla of the descending part and showed that the duodenum was enlarged. Cannulation was performed through the duodenum to the main pancreatic duct, and the guide wire was introduced into the pancreatic duct (Figures 2A,B). When 38% of the contrast agent (meglumine diatrizoate) was injected, stones with a maximum diameter of approximately 1.5 cm * 1.2 cm were observed, multiple filling defects in the pancreatic duct and evident narrowing of the pancreatic duct at the head of the pancreas (Figure 3A). Endoscopic sphincterotomy (EST) and columnar balloon dilatation were performed for 30 s (Figure 3B). Subsequently, after cylindrical balloon dilation was accomplished, SpyGlass was inserted under a guide wire's guidance (Figure 4A–D). Furthermore, the insertion of the imaging catheter into the pancreatic duct through the SpyGlass active channel and sterile water (water for injection) into the pancreatic duct was executed to make the surgical field clear. The SpyGlass imaging catheter was pushed to the pancreatic duct to observe the stone shape, size and presence of incarceration under direct vision. White stones blocking the trunk of the pancreatic duct were seen, and the insertion of the U100 Plus EHL lithotripsy fibre through the working pipe to the stone's surface for Electrohydraulic lithotripsy was carried out, which allowed the large stones to be crushed (Figures 4E,F). Irrigation was performed through a dedicated channel, and the pancreatic duct was cleaned with a dilation balloon (Figures 5A,B). Following the placement of a pancreatic duct stent [5F × 9 cm, Micro-Tech (Nanjing) Co. Ltd.] was performed, marking the end of the entire operative procedure (Figure 5C,D). Intraoperative oxygen inhalation, oxygen saturation monitoring, and oxygen saturation of approximately 95%–99% were applied during the entire procedure.

At the end of the operation, keeping the nothing by mouth (NPO), symptomatic treatment, including fluid replacement, acid suppression and anti-infection, was carefully performed. Postoperative infusion of somatostatin and rectal application of indomethacin suppositories were performed to prevent post-ERCP pancreatitis(PEP). Observing the patient's condition and symptoms, such as fever, abdominal pain, abdominal distension, hematemesis and melena, were monitored. Re-examination 24 h after operation: leukocytes 5.75 × 10^9^/L, blood amylase (U/L) 130, procalcitonin (pg/ml) 0.351; 48 h after the operation, blood amylase (U/L) 83, pancreatic juice culture results were negative. Abdominal coloured Doppler ultrasound was performed two days after the operation and showed that the indwelling tube was visible in the main pancreatic duct with echo reduction and that no residual stones were observed. Even more encouraging was the patient's absence of harmful side effects during treatment. The patient's blood amylase, urine amylase and infection indexes have returned to normal range, and the general condition has improved significantly. Pancreatic juice cultures were negative. After discharge, patients were regularly followed up on the smooth drainage of pancreatic duct stents, two months later, the pancreatic stent was successfully removed under endoscopy with a snare, and no residual stones were found by ultrasound.

## Discussion

### Overview of PDS

In recent years, as people's living standards in developing countries have generally improved, the incidence of pancreatic duct stones has gradually increased (6). The main etiologies and risk factors include alcoholism, idiopathic, cholelithiasis-related, autoimmune pancreatitis, complications after pancreaticoduodenectomy, genetic or familial factors, hyperthyroidism, and others (7). According to the research of Inui K et al. (8), the highest proportion of pancreatic duct stones caused by alcohol drinking in men was 73.2%, while 34.9% of women were idiopathic. On the other hand, PDS and CP can be causal to each other. Pancreatic inflammation leads to the formation of pancreatic duct stones, which obstruct the pancreatic duct, causing stenosis and dilation of multiple pancreatic ducts (9). PDS can result in obstruction, leading to increased pancreatic duct pressure, aggravation and destruction of pancreatic tissue structure, as well as impairment of the internal and exocrine functions of the pancreas. In addition, studies have shown that if patients with PDS are not diagnosed and treated in time or have repeated stones, they may even lead to the malignant progression of pancreatic cancer (10). Therefore, it is essential to follow individualized treatment on time.

### From ERCP to SpyGlass

The treatment measures of PDS mainly include surgical treatment, extracorporeal shockwave lithotripsy (ESWL) and endoscopic treatment (11). In the past, surgery was often used to treat PDS. It usually involves an incision of the pancreatic parenchyma to find the pancreatic duct and removal of the stone for pancreaticojejunostomy (12). Although surgical treatment is similar to endoscopic treatment in terms of clinical relief, surgical treatment is invasive and is associated with many complications. With the development and improvement of therapeutic endoscopy technology, endoscopic treatment has achieved good results and demonstrated its advantages of safety, minimal invasiveness and high efficiency (13). It further avoids the high risks of surgical operations, prominent trauma, slow recovery, many complications, and postoperative effects on the endocrine function of the patient's pancreas (14). The development of ERCP has led to a new era for the diagnosis and treatment of biliary and pancreatic diseases (15). Although ERCP technology has been developed and perfected, it remains limited by the fact that the endoscopic specialist is only able to visualize structures indirectly *via* fluoroscopy. This indirect visualization can be especially limited in patients with larger pancreatic duct stones and indeterminate pancreatic duct strictures (16, 17). In addition, the diameter of the pancreatic duct is minimal, and ERCP is difficult for some stone fragments to be extracted entirely and removed, including stones distributed in the tail of the pancreatic duct, pancreatic duct stricture and large stones embedded in the pancreatic duct (18). The ERCP shortcomings, on the other hand, include: ①The presence of blind spots in the exploration of the biliary and pancreatic ducts makes the imaging of the biliary and pancreatic ducts possible only by indirect angiography, while the lesions cannot be directly judged. ②The low stone retrieval rate and the therapy for complex stones are inefficient, especially for stones distributed in the tail of the pancreatic duct combined with narrow pancreatic ducts and large stones, as well as stones embedded in the pancreatic duct. ③ERCP has a low positive rate of cell brushing and scratching of lesions and is often challenging to meet the needs of clinical diagnosis and treatment.

In our case study, the utilization and incorporation of the SpyGlass examination provided better visualization of the pancreatic duct tree, helping to determine the extent of pancreatic duct injury and prognosticate the disease's outcome. This procedure is acknowledged as a safe and effective method for evaluating and managing complex pancreatic duct stones and indeterminate pancreatic duct strictures (19). The advantages of SpyGlass can be summarized as follows: ①the procedure allows direct access into the pancreatic duct lumen and improves the success rate of stone retrieval under direct vision. Therefore, it appears to be the best diagnostic method for the early detection of minimal pancreatic lesions (20). ②This direct biopsy has high accuracy. Furthermore, for patients suspected of pancreatic duct tumours, the pathological diagnosis can be made by direct observation of the lumen and biopsy through SpyGlass (21). ③The SpyGlass technique further increases the target tissue location for complicated pancreatic duct stones, and it is possible to guide intracavitary laser lithotripsy to successfully remove the stone, avoiding the risk of surgical incision and stone removal (22). ④It also overcomes the need for two surgeons to operate and cooperate at the same time together with an endoscopist who needs to perform pancreatic duct laser lithotripsy and electrohydraulic lithotripsy under direct vision (23). ⑤This technique further provides a 360° panoramic view of the lumen and is convenient for placing a pancreatic duct stent in the narrow part to reduce obstruction (24). Given that, these advantages cover the deficiencies of traditional ERCP technology.

### Comparison of intraductal therapies and ESWL

The European Society of Gastrointestinal Endoscopy (ESGE) suggests endoscopic therapy and/or mechanical ESWL be the first-line treatment for painful uncomplicated chronic pancreatitis with an obstructed main pancreatic duct in the head/body of the pancreas. Among them, ESWL utilizes a confined space to release shock waves instantly, smashes pancreatic duct stones *in vitro*, and achieves the purpose of clearing stones through spontaneous expulsion or subsequent ERCP. A retrospective cohort study by Benjamin L et al. (25) showed that 240 patients were treated with ESWL, and 18 were treated with single-operator pancreatoscopy with intraductal (intracorporeal) lithotripsy (SOPIL). The overall technical success rate of stone clearance was 224/258 (86.8%), which was similar between the ESWL and SOPIL groups (86.7% vs. 88.9%, *p* = 1.000). A SOPIL approach required fewer total procedures (1.6 ± 0.6 vs. 3.1 ± 1.5, *p* < 0.001) and less aggregate procedure time (101.6 ± 68.2 vs. 191.8 ± 111.6 min, *p* = 0.001). Adverse event rates were similar between the groups (6.3% vs. 5.6%, *p* = 1.000). The use of SOPIL was independently associated with greater efficiency compared to ESWL [OR 5.241 (1.348–20.369), *p* = 0.017]. Stone size > 10 mm was associated with less efficient stone clearance [OR 0.484 (0.256–0.912), *p* = 0.025]. Given that, ESWL and SOPIL are safe and effective endoscopic adjunct modalities for treating large pancreatic duct stones. SOPIL is an emerging alternative to ESWL that is potentially more efficient for lithotripsy and MPD stone clearance. Major complications of ESWL include perirenal hematoma, pancreatic duct obstruction, intestinal perforation, spleen rupture, pulmonary trauma, and necrotizing pancreatitis (26). The success of extracorporeal lithotripsy has a certain relationship with the hardness of the stone, and the recurrence rate of stones in those who successfully removed pancreatic duct stones with ESWL is 18% to 22% (27). Given the above situation, we use the SpyGlass lithotripsy technique under direct vision to get closer to the stone itself, break it, and then remove the pancreatic duct stone through the stone extraction balloon.

In recent years, Per-oral panereatoscopy (POP)-guided lithotripsy (SpyGlass) has been used in the treatment of pancreatic duct stones, which is mainly combined with EHL or LL for lithotripsy (17, 23). Although both EHL and LL attempt to fragment pancreatic duct stones, achieving this is fundamentally different between the two strategies. EHL uses high-amplitude shock wave lithotripsy generated by two coaxial insulated electrodes to perform fixed-point micro-blasting on the stone. However, the strong impact and high heat accompanying hydroelectric lithotripsy may damage the pancreatic duct, and the appearance of the pale area in the visual field of the lithotripsy will also affect the operation (28). LL uses water, pancreatic juice and blood as the medium, emits it in a pulsed manner and generates laser light through the optical fibre. It has the advantages of low current hot spot release and small tissue penetration depth, and its penetration depth into tissue is shallow, and it is not easy to damage the pancreatic duct. The disadvantage is that the optical fibre may be bent and broken, resulting in treatment failure, and the inaccurate power setting will also cause damage to the pancreatic duct tissue (29). A recent study included 370 patients, of whom 218 were treated with EHL and 155 with LL. The overall POP pooled technical and clinical success rates were 88.1% and 87.1%. For EHL-POP, the pooled technical success rate was 90.9% (95% CI 87.2%–95.2%), and the pooled clinical success rate was 89.8% (95% CI 87.2%–95.2%). While for LL-POP, the pooled technical and clinical success rate was 88.4% (95% CI 85.9%–95.1%) and 85.8% (95% CI 80.6%–91.6%). In total, 43 adverse events occurred (12.1%;95% CI 8.7%–15.5%) (30). Another recent meta-analysis of pancreatic duct stone management by McCarty et al. showed that in 302 patients, Pooled technical success was 91.18%, with an overall fragmentation success of 85.77%. Single lithotripsy session stone fragmentation and pancreatic duct clearance occurred in 62.05% of cases. Comparing POP-EHL vs. POP-LL, there was no significant difference in technical success, fragmentation success, single session duct clearance, or adverse events (*p* > 0.0500). This systematic review and meta-analysis demonstrated there was no statistical difference based on performance measures (17). So it follows that technical success, fragmentation success, single session duct clearance, and adverse events were not different between EHL and LL, as evidenced by the overlapping confidence intervals.

There are a series of case reports on treating PDS with spyglass endoscopy (Table 1). Relevant clinical studies pointed out that ERCP combined with SpyGlass system laser lithotripsy was very successful in treating pancreatic duct stones. The complication rate is not significantly different from conventional endoscopic retrograde cholangiopancreatography. Therefore, SpyGlass appears to be a safe and effective tool in routine clinical practice. In addition, this study has some limitations, including the fact that only one case was evaluated, making it necessary to test this treatment strategy in more cases. More clinical trials and high-quality studies are needed to confirm the efficacy and safety of this regimen in treating patients with PDS.

**Table 1:** Treatment of PDS with SpyGlass under the guidance of endoscopy.

Year/author	Nation	type	Approach	Number of reported cases	Stone removal rate	Technical success rate	Complication rate
2016/Navaneethan et al (20)	US	PDS	LL	5	4 (80%)	100%	20%
2017/ Bekkali et al (21)	UK	PDS	EHL	6	6 (100%)	100%	1.2%
2019/Ogura et al (22)	Japan	PDS	EHL	21	18 (85.7%)	100%	4.7%
2019/Brewer et al (23)	US, Europe	PDS	EHL/LL	109	98 (89.9%)	84%	10.1%
2019/Gerges et al (24)	Germany	PDS	EHL/LL	20	19 (95%)	95%	30%

## Conclusion

The association of SpyGlass with the ERCP procedure in our case study proved feasible, performed safely, and provided a detailed evaluation of injured pancreatic ducts. It further added elements to determine the disease prognosis and provided a good plan for future management. Our results prove that LL or EHL combined with SpyGlass endoscopy under direct vision can significantly improve the success rate and safety of treatment in such cases and is worthy of clinical promotion. In addition, When PDS occurs, the patient's condition should be fully grasped in time to avoid delaying the disease. Using imaging, ERCP, SpyGlass and other methods to achieve complementary advantages and precise individualized treatment can reduce the pains of patients without affecting the treatment effect, at the same time, reduce the recurrence rate and complications of PDS and maximize the benefits of patients. At the same time, insisting on regular follow-up after surgery is also the focus of improving the treatment effect of such patients. Endoscopic combined therapy is a minimally invasive, safe and effective diagnosis and treatment method. With the continuous development and improvement of endoscopic treatment technology, it will undoubtedly continue to write a new chapter in diagnosing and treating hepatobiliary and pancreatic diseases.

## Data Availability

The original contributions presented in the study are included in the article/Supplementary Material, further inquiries can be directed to the corresponding author/s.
